# Impact of panelists’ experience on script concordance test scores of medical students

**DOI:** 10.1186/s12909-020-02243-w

**Published:** 2020-09-17

**Authors:** Olivier Peyrony, Alice Hutin, Jennifer Truchot, Raphaël Borie, David Calvet, Adrien Albaladejo, Yousrah Baadj, Pierre-Emmanuel Cailleaux, Martin Flamant, Clémence Martin, Jonathan Messika, Alexandre Meunier, Mariana Mirabel, Victoria Tea, Xavier Treton, Sylvie Chevret, David Lebeaux, Damien Roux

**Affiliations:** 1 Department of Emergency Medicine, Saint-Louis University Hospital, Assistance Publique-Hôpitaux de Paris, 1 avenue Claude Vellefaux, 75010 Paris, France; 2 SAMU de Paris, SMUR Necker, Necker Enfants Malades University Hospital, Assistance Publique-Hôpitaux de Paris, Paris, France; 3 Department of Emergency Medicine, SMUR, Lariboisère University Hospital, Assistance Publique-Hôpitaux de Paris, Paris, France; 4 Paris Diderot University, Paris, France; 5 Department of Pneumology, Reference Center for Rare Pulmonary Diseases, Bichat University Hospital, Assistance Publique-Hôpitaux de Paris, Paris, France; 6 INSERM, UMR 1152, Paris Diderot University, Paris, France; 7 Department of Neurology and Stroke Unit, Sainte-Anne University Hospital, Paris, France; 8 INSERM, UMR 1266, Psychiatry and Neurosciences Institute of Paris, Paris-Descartes University, Paris, France; 9 Paris-Descartes University, Paris, France; 10 Department of Getriatric Medicine, Louis-Mourier University Hospital, Assistance Publique-Hôpitaux de Paris, F-92700 Colombes, France; 11 INSERM, UMR 1132, BiOsCar, University of Paris, Paris, France; 12 Department of Kidney Physiology, Bichat University Hospital, Assistance Publique-Hôpitaux de Paris, Paris, France; 13 INSERM, UMR 1149, Inflammatory Research Center, Paris, France; 14 University of Paris, Paris, France; 15 Department of Respiratory Medicine, Cochin University Hospital, Assistance Publique-Hôpitaux de Paris, Paris, France; 16 Cochin Institute, UMR 1016. Paris-Descartes University, Paris, France; 17 Pulmonology and Lung Transplant Unit, Bichat University Hospital, Assistance Publique-Hôpitaux de Paris, Paris, France; 18 Physiopathology and Epidemiology of Respiratory Diseases (PHERE), INSERM, UMR 1152, and Paris Transplant Group, Paris, France; 19 Department of Cardio-oncology, Georges Pompidou European University Hospital, Assistance Publique-Hôpitaux de Paris, Paris, France; 20 INSERM, UMR 970, Paris Cardiovascular Research Center PARCC, Paris, France; 21 Department of Cardiology, Georges Pompidou European University Hospital, Assistance Publique-Hôpitaux de Paris, Paris, France; 22 Department of Gastroenterology, Inflammatory Bowel Disease, and Nutritive Assistance, Beaujon University Hospital, Assistance Publique-Hôpitaux de Paris, Clichy, France; 23 Department of Biostatistics and Medical Information, Saint-Louis University Hospital, Assistance Publique-Hôpitaux de Paris, Paris, France; 24 Centre of Research in Epidemiology and StatisticS (CRESS), INSERM, UMR 1153, Epidemiology and Clinical Statistics for Tumor, Respiratory, and Resuscitation Assessments (ECSTRRA) Team, University of Paris, Paris, France; 25 Department of Microbiology, Mobile Infectiology Unit, Georges Pompidou European University Hospital, Assistance Publique-Hôpitaux de Paris, Paris, France; 26 Department of Intensive Care, Louis Mourier University Hospital, Assistance Publique-Hôpitaux de Paris, F-92700 Colombes, France

**Keywords:** Script concordance test, Medical student, Panelist

## Abstract

**Background:**

The evaluation process of French medical students will evolve in the next few years in order to improve assessment validity. Script concordance testing (SCT) offers the possibility to assess medical knowledge alongside clinical reasoning under conditions of uncertainty. In this study, we aimed at comparing the SCT scores of a large cohort of undergraduate medical students, according to the experience level of the reference panel.

**Methods:**

In 2019, the authors developed a 30-item SCT and sent it to experts with varying levels of experience. Data analysis included score comparisons with paired Wilcoxon rank sum tests and concordance analysis with Bland & Altman plots.

**Results:**

A panel of 75 experts was divided into three groups: 31 residents, 21 non-experienced physicians (NEP) and 23 experienced physicians (EP). Among each group, random samples of *N* = 20, 15 and 10 were selected. A total of 985 students from nine different medical schools participated in the SCT examination. No matter the size of the panel (N = 20, 15 or 10), students’ SCT scores were lower with the NEP group when compared to the resident panel (median score 67.1 vs 69.1, *p* < 0.0001 if N = 20; 67.2 vs 70.1, *p* < 0.0001 if *N* = 15 and 67.7 vs 68.4, *p* < 0.0001 if *N* = 10) and with EP compared to NEP (65.4 vs 67.1, *p* < 0.0001 if N = 20; 66.0 vs 67.2, *p* < 0.0001 if N = 15 and 62.5 vs 67.7, *p* < 0.0001 if N = 10). Bland & Altman plots showed good concordances between students’ SCT scores, whatever the experience level of the expert panel.

**Conclusions:**

Even though student SCT scores differed statistically according to the expert panels, these differences were rather weak. These results open the possibility of including less-experienced experts in panels for the evaluation of medical students.

## Background

The evaluation process of medical students before they become certified doctors is a major educational challenge. This assessment should evaluate medical knowledge but also competencies such as clinical reasoning. Multiple choice questions accurately assess theoretical medical and scientific knowledge [1], but decision-making skills required in medicine are much more complex to evaluate [2]. Developing adequate clinical reasoning skills is crucial for the management of patients and must be properly evaluated.

Script concordance testing (SCT) is a validated method that offers the possibility to assess medical knowledge alongside clinical reasoning under conditions of uncertainty [3, 4]. This assessment tool was designed with the cognitive psychology script theory in order to objectively examine if the students’ knowledge is organized for clinical decision-making [3]. SCT is designed to assess a candidate’s reasoning skills with the challenging decisions that are encountered during real-life clinical practice for diagnosis, investigation or treatment of patients [3, 4].

SCT follows a well-defined plan [5]: students are challenged with a clinical case, a hypothesis (diagnosis, prescription, etc.…) is proposed and followed by an additional finding. Students have to determine how this new information modifies their hypothesis through a Likert scale representing the influence of this additional information on their medical reasoning (from “-2” to “+ 2”). The scoring, which is based on the aggregate scoring method, takes into account the variability of the clinical reasoning process among a panel of experts. Previous studies using SCTs have confirmed the reliability and validity of this evaluation method [5–8], and clinical experience could be associated with improved SCT results [5]. SCTs could therefore encourage medical students to focus on their bed-side training in addition to the traditional curriculum. However, a study previously highlighted the difficulties in recruiting and training expert panelists across all medical disciplines to ensure a sufficient number of high quality SCTs [9]. The ideal number and type of experts to form an expert panel also remains an unanswered question. According to the literature review by Dory et al the size of the expert panel should lie between 10 and 20 [10]. Charlin et al showed that students’ SCT scores were higher when the panel was composed of teaching experts than with non-teaching experts, but ranking provided by the two panels was similar [11].

In this study, we compared the students’ SCT scores according to the different experts’ professional experience and the number of experts in panels. We also explored the relationships between the SCT scores and the students’ previous clinical training.

## Methods

### Construction of the test

This work was conducted by a group of 20 physicians and medical students involved in medical education and evaluation at the medical schools of Paris Diderot and Paris Descartes Universities, France. The first step was to create SCTs according to the SCT construction guidelines as described by Fournier *et al* [12]. The working group was divided into six subgroups which each created five to eight SCTs in the fields of cardiology and emergency medicine, in order to correspond to the students’ 5th year curriculum. SCTs were then reviewed by the whole working group and those with too much or too little variability among the responses were removed. Thus, 27 SCTs with a total of 104 items were validated, covering diagnosis, investigation and treatment.

### Reference panel construction

An expert panel of cardiologists and emergency physicians were asked to answer SCTs through GoogleForms® questionnaires (Google©, Mountain View, CA). Before answering the test, experts were asked to fill out a form including the following characteristics: their specialty, academic degree (resident, non-teaching or teaching functions) and the number of years of their clinical experience after residency. Each specialty panel received at least three invitations to fill in the questionnaire.

### Development and selection of SCT questions

The group performed a final review of the SCTs and selected nine SCTs with a total of 30 items in order to construct a one-hour SCT exam. Those with too much variability among expert answers were removed. Also, a “balanced approach” was favored to achieve a balance between items with extreme responses (anchors “-2” and “+ 2”) and those with median responses (anchor “0”) in order to mitigate the SCT scores of the low-performing students who had previously been shown to only select median responses and avoid the extreme responses [13].

### Scoring process

The experts’ answers were used to build the correction key, with the methodology described by Gagnon *et al* [6]. Students received one point when they chose the modal answer (that is to say, the anchor that was chosen by the largest number of experts), zero points when their answer was not chosen by any expert and a proportional partial point when their answer was chosen by some experts but not the majority of them. The process consisted of dividing all answers for an item by the modal number of experts for that item. For example, if 17 experts (out of 20) had chosen the anchor “-1″ and three the anchor “0″, then “-1″ received one point (17/17), “0″ received 0.18 (3/17) and other anchors received zero. The total score for the test was the sum of points obtained for each item. Numbers were then scaled to get a maximum of 100. Students’ scores were calculated according to different panels of experts, depending on the number of experts, specialty, and experience.

### Candidates

A group of 2312 undergraduate medical students in the 5th year of their medical studies at the Universities of Besançon, Lorraine, Versailles Saint-Quentin, Créteil-Paris Est, Paris Diderot, Paris Descartes, Paris Sorbonne Université, Paris Sud (France) were asked to complete the computer-based SCT as an optional session during a mock national ranking exam [14]. The SCT exam was administered through the online national evaluation system of French medical school [15]. At the beginning of the SCT exam, students were asked if they had previously performed a traineeship in cardiology or emergency medicine. A brief explanation was given to the students before the beginning of the exam and the first SCT was considered as SCT training. SCT examination scores were immediately given to the students but were not included in their final year examination score. Immediate feedback was made available to participating students with global answers by experts and detailed comments by two individual experts for each item as proposed by Fernandez *et al* [16]. In France, all undergraduate students have similar curricular plans, teaching staff availability, learning methodologies and assessment formats which prepare them for the same national final exam.

### Endpoints

We aimed to determine the effect of the panel of experts’ clinical experience and specialties on students’ SCT scores by comparing these scores obtained with each type of panel. Because SCT assesses real-life clinical practice reasoning skills and is supposed to favor students who have completed a traineeship, our secondary endpoint compared SCT examination scores of students whether they had already performed a traineeship in the concerned specialty or not.

### Statistical analysis and ethics

Results are reported as median with interquartile range (IQR) for continuous variables and number with percentage for binary and categorical variables. SCTs with five or more missing items out of 30 were excluded.

The experts were divided into three groups depending on their professional experience and, for non-resident physicians, based on their post-residency years of experience, distinguished as “non-experienced physicians” if they had less experience after residency and “experienced physicians” if they had more than a certain year of experience after residency. The time cut- off was the median years of experience after residency in the sample of experts. In order to compare equivalent panels, and because the ideal number of experts to form an expert panel remains an unanswered question but stands between 10 and 20 [6], a random sample of 20, 15 and 10 out of each group was selected once.

Reliability of scales was estimated with Cronbach’s alpha coefficient, calculated for each series of scores, depending on the level of experience. Student scores, obtained with each panel of experts, were compared with a paired sample Wilcoxon rank sum test. In order to assess concordance between student scores depending on the panel of experts, Bland & Altman graphics were plotted. SCT scores of students who had previously performed a traineeship in cardiology or emergency medicine were compared to those of students who had not performed a traineeship. This last comparison was not performed at last. For these comparisons, we used SCT scores obtained by students with the panel of experienced physicians.

All *p*-values were two-sided, with values of 0.05 or less considered as statistically significant. Data were analyzed with R 3.5.0 software (the R Foundation for Statistical Computing, Vienna, Austria).

This teaching project was validated and authorized by the Paris Descartes University teaching board. Students were informed orally at the beginning of the SCT exam that this study was being conducted. There were no participation incentives. No written consent was requested by the Paris Descartes University teaching board for this study.

## Results

**Fig. 1:**
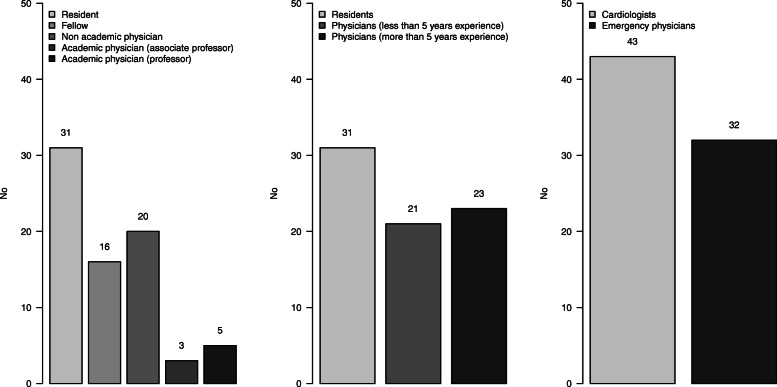
Number of experts according to their academic degree (left), clinical experience (middle) and specialty (right)

A total of 108 experts answered the SCT, including 33 with incomplete forms. Thus, the expert panel consisted of 75 experts, including 43 cardiologists and 32 emergency physicians. Among non-resident physicians, median experience was 5 [2-12] years, ranging from zero to 30 years (Additional file 1). The panel was divided into three groups: 31 residents, 21 non-experienced physicians and 23 experienced physicians (Fig. 1).

Among the 2312 students who were invited to participate in the SCT examination, a total of 985 (42.6%) answered SCTs. After excluding 50 students with at least five unanswered items out of the 30 on the SCT, we retained 935 students for final analysis.

Additional file 2 shows the repartition of the answers on the Likert scale (“-2” to “+ 2”) given by the whole panel of experts. Among the 2250 answers given by the 75 experts to the 30 items, answers to the SCT were rather balanced for mid answers (524 answered “-1”, 668 answered “0” and 664 answered “+ 1”) but with fewer extreme answers (243 answered “-2” and 147 answered “+ 2”).

Cronbach’s alpha coefficients ranged from 0.43 to 0.53 depending on the panel of experts and the sample size of the panel, illustrating an acceptable internal consistency (Additional file 3) [17].

**Fig. 2:**
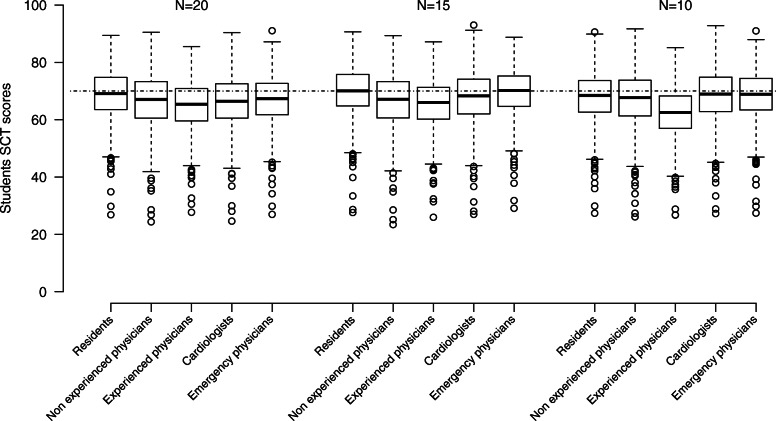
Script concordance testing (SCT) scores of the 935 medical students on a scale from zero to 100 according to the level of experience (residents, non-experienced and experienced physicians), the specialties (cardiologists and emergency physicians) and the size (*N* = 20, 15 and 10) of the panel of experts. Dotted line corresponds to an STC score of 70

Figure 2 and Additional file 4 show the SCT scores according to the level of experience, the specialties (cardiologists vs emergency physicians) and the sample size (20, 15 and 10) of the panel of experts. No matter the size of the panel, students’ median SCT scores were lower when the panel of experts were non-experienced physicians compared with residents (67.1 vs 69.1, *p* < 0.0001 if *N* = 20; 67.2 vs 70.1, *p* < 0.0001 if *N* = 15 and 67.7 vs 68.4, *p* < 0.0001 if *N* = 10) and lower with experienced physicians compared to non-experienced physicians (65.4 vs 67.1, *p* < 0.0001 if *N* = 20; 66.0 vs 67.2, *p* < 0.0001 if N = 15 and 62.5 vs 67.7, *p* < 0.0001 if *N* = 10).

**Fig. 3:**
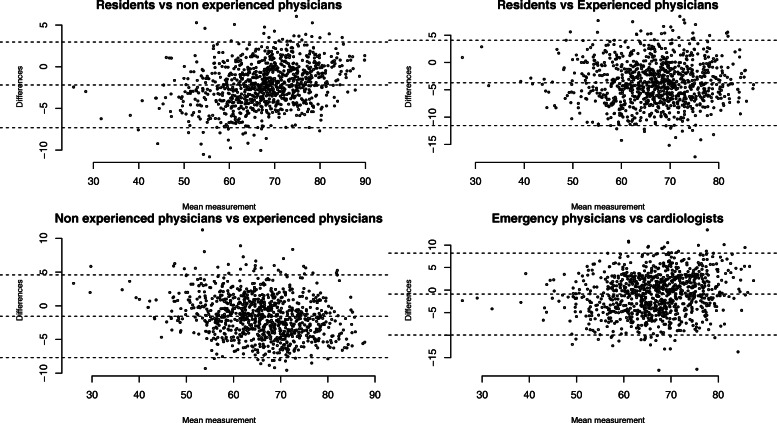
Concordance between students’ Script concordance testing (SCT) scores depending on the panel of experts with Bland & Altman graphics. Each dot shows the difference between two SCT scores for a student depending on the panel of experts (**a**: resident and non-experienced physicians, **b**: residents and experienced physicians, **c**: non-experienced physicians and experienced physicians and **d**: emergency physicians and cardiologists) over the mean of these two scores. For each graphic, dotted lines are the mean of the differences and 2 standard deviations (SD) above and below that. The mean of the differences and 2 SD were − 2.18, − 7.31, and 2.95 for graphic **a**, − 3.73, − 11.53 and 4.08 for graphic **b**, − 1.55, − 7.68 and 4.58 for graphic **c** and − 0.88, − 9.97 and 8.20 for graphic **d** showing small differences between scores, whatever the experience or specialty of the panel of experts

As shown on Fig. 2 and Additional file 5, the comparison amongst specialties revealed that student median SCT scores were lower when using the cardiologist panel compared with the emergency physicians panel (66.4 vs 67.3, *p* < 0.0001 if *N* = 20 and 68.3 vs 70.2, *p* < 0.0001 if *N* = 15) except for the panel of 10 experts (68.9 vs 68.9, NS). However, Fig. 3 shows good concordance between student SCT scores, whatever the experience or the specialty of the panel of experts, according to Bland & Altman plots.

**Fig. 4:**
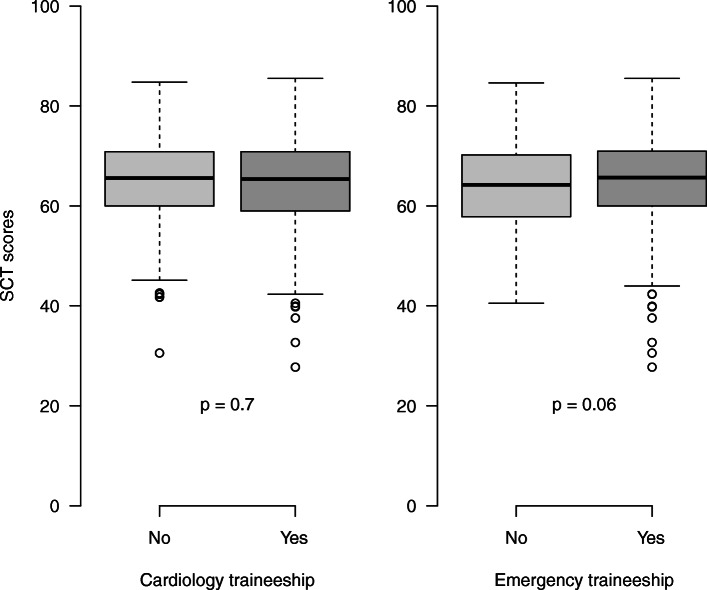
SCT scores depending on having done or not a traineeship in cardiology or emergency medicine

Among the 935 students who participated in the SCT examination, 390 had performed a traineeship in cardiology and 728 in emergency medicine and 87 had not performed any traineeship in these medical departments. Figure 4 shows student SCT scores according to the existence or absence of previous traineeship. The comparison of SCT scores depending on traineeship evidenced no difference.

Additional file 6 shows student SCT scores depending on the number of traineeships performed by students: 87 students had not yet performed any traineeship in cardiology or emergency medicine; 538 students had performed one traineeship and 310 had performed both. There was no significant difference depending on the number of traineeships.

## Discussion

This study reports the first real large scale Concordance Test for undergraduate medical students at multiple universities in France. Our study aimed at exploring the relationships among expert specialties of the panelists, previous clinical training of students and SCT results. We first compared the students’ scoring to the different expert panels with varying levels of clinical experience. We found a significant difference with lower SCT scores for students when experts had greater experience. However, the absolute differences in scores were small. This may be related either to the fact that experts considered as “experienced” in our panel had a median experience of only 5 years, or to the low difficulty level of the SCT specifically developed for undergraduate students. This highlights the fact that the composition of the expert panel is perhaps not that relevant when evaluating undergraduate medical students. This is encouraging since it has been difficult to recruit enough experts with clinical experience to participate in the panels. The existing literature illustrates a correlation between previous clinical experience of students and SCT scores. For example, for postgraduate students, a linear progression of scores with increasing clinical experience has been reported [18]. We did not observe better scores for students who had done a rotation in a cardiology or an emergency department. It is possible that our selection of SCT was too “generalist” and therefore failed to discriminate between the specialized past training. It confirms the dictum that what is asked is more important than how it is asked. As a matter of fact, all students spent a similar amount of time in clinical wards (emergency department, cardiology or others such as pneumology, critical care, etc.…). Our SCT questions were developed specifically for use in assessment of undergraduate medical students. SCTs considered as too specialized were indeed voluntarily removed from the exam.

One of the major strengths of our study was the condition of the test which was administered similarly to the official national ranking exam, and therefore in real-life settings. The students did not have the possibility to consult resources or exchange information with other candidates during the test. This strengthens the validity of our results. Many other SCT assessments have been conducted in other settings such as web-based [4–7]. The overall number of participants in the expert panels and the students is satisfactory, being to our knowledge the largest cohort of undergraduate medical students.

Another strength of our study is the balanced design of our SCT. Previous studies have raised the issue that a medical student’s use of the strategy of avoiding extreme answer options in SCTs may potentially have an impact on the validity of the test results [13]. We designed “balanced” SCTs in order to insure the validity of our test. The real-life SCT exam took place during a mock session to prepare students for the national ranking exam of the French medical curriculum. The SCT exam was optional, so we can question the possibility that some students could have used strategies to score higher results, as this was not a qualification exam.

It would be interesting to understand the process associated with SCT for students to insure effective learning. The think-aloud method has been proposed to allow students to justify their reasons for choosing a particular response option in answering the SCT. Some authors have suggested ‘think-aloud’ might help to shed further light on examinees’ use of probability versus typicality-based reasoning strategies in responding to SCT items [18].

The necessary steps to reach the large-scale SCT were complex and arduous. This raises the question of the acceptability of such a paradigm change in French medical education. The reasons to explain these difficulties are numerous. First, even though this educational format has been largely validated and is already used in other countries, the majority of French educators and students are not familiar with the SCT format. It was therefore difficult to obtain the participation of the required experts for the validation of our SCT. French academic professors are professionals linked to numerous medical specialties. It was also difficult to identify 15 practitioners for each panel who were not overly sub-specialized and therefore could be used as a reference panel. We decided therefore to create a reference panel of “experienced” members, based on their professional experience and their specialty rather than their academic title. One of the reasons for the poor participation rate from the solicited experts is likely related to the excessive workload in academic medicine. In France, being an academic physician implies working in clinical practice but also conducting research projects and teaching as a professor for the medical faculty. Some studies on SCT have described the high prevalence of burnout amongst the population of experts [9]. To avoid burnout in experienced, volunteer members of expert reference panels, various strategies have been evoked such as sharing work with affiliated universities, replacing single discipline panels with multidisciplinary panels, and hiring recent medical graduates as members of the expert panel [9]. This was a major issue regarding the feasibility of integrating routine SCT testing in our faculties.

Lastly, the responses, though marked objectively, are actually based on expert subjective judgment. However, the importance of the objectivity in the assessment of medical students has been recently challenged in the context of competency-based education. A part of subjectivity should probably be accepted since it is not only unavoidable but also necessary as soon as assessment is based on expert judgment [19].

### Limitations

One of the major limitations of our work is the low number of highly experienced physicians. This could explain the small difference between the students’ scores depending on the experts’ experience and illustrates the current demography in academia. However, the weak differences in scoring between the different expert panels raised the question of the importance of experience in creating and scoring SCTs for undergraduate medical students. The possibility of having postgrads as experts has already been studied [9]. Our results confirm this may be a solution to insuring the feasibility of the SCT as a routine assessment method in French medical schools. Another limitation is that we only performed one sampling of *N* = 10, *N* = 15 and *N* = 20 among each group of experts. It is possible that a different sampling would have given different results. It would have been interesting, as Gagnon et al did in their work [6], to generate several random panels of reference of size 10, 15 and 20, and compare the SCT scores accordingly. However, the limited number of experts in some groups (21 non-experienced physicians) would not have allowed enough combinations.

## Conclusions

Our study showed significant differences between student SCT scores according to the expert panels. This difference, however, seemed weak. Therefore, when developing SCTs to evaluate medical students, the level of experience of the experts has only a small impact on students’ scores. This observation allows the recruitment of less-experienced experts and thus improves the feasibility of the SCT for routine use for undergraduate medical students.

## Supplementary information

**Additional file 1.** Years of clinical experience after residency for non-resident physicians.

**Additional file 2.** Distribution of the 75 experts’ answers on the Likert scale of the 30 items.

**Additional file 3.** Cronbach’s alpha coefficients according to the experience, the specialty and the sample size of the panel of experts.

**Additional file 4 **SCT scores of the 935 medical students according to the level of experience (residents, non experienced and experienced physicians) and the size (*N* = 20, 15 and 10) of the panel of experts.

**Additional file 5.** SCT scores of the 935 medical students depending on the specialties (cardiologists and emergency physicians) and the size (N = 20, 15 and 10) of the panel of experts.

**Additional file 6.** SCT scores depending on the number of traineeships performed by students in cardiology or emergency medicine.

## Data Availability

The datasets during and/or analyzed during the current study are available from the corresponding author on reasonable request.
