# Supplementary figures and images for: Impact of panelists’ experience on script concordance test scores of medical students
Source: BMC Med Educ. 2020 Sep 17;20:313. doi: 10.1186/s12909-020-02243-w (PMC7499961; doi:10.1186/s12909-020-02243-w)

**Additional file 1** Years of clinical experience after residency for non-resident physicians

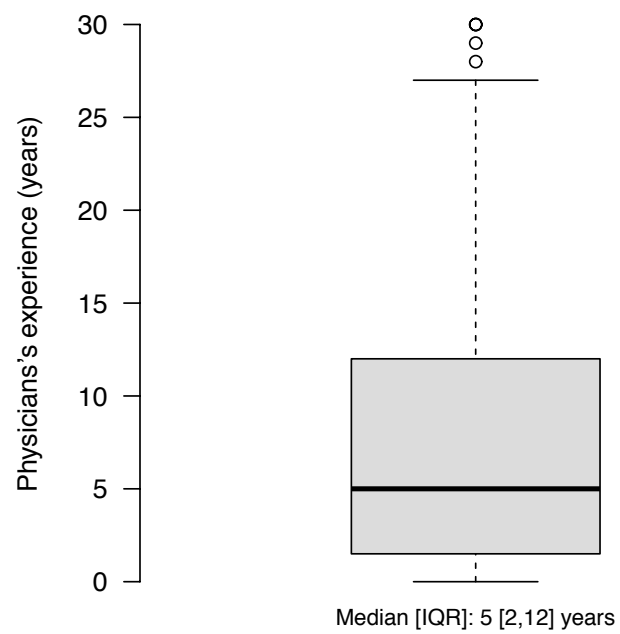

Supplement: Supplementary file 1 — Additional file 1. Years of clinical experience after residency for non-resident physicians. [file 12909_2020_2243_MOESM1_ESM.pdf]

**Additional file 2** Distribution of the 75 experts' answers on the Likert scale of the 30 items

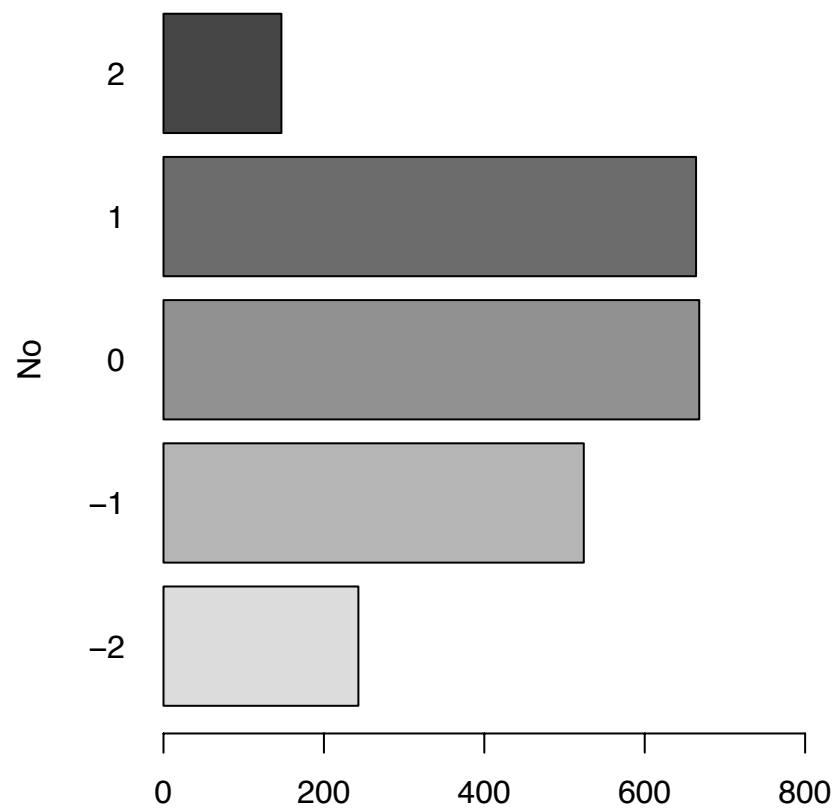

Supplement: Supplementary file 2 — Additional file 2. Distribution of the 75 experts’ answers on the Likert scale of the 30 items. [file 12909_2020_2243_MOESM2_ESM.pdf]
