# Supplementary material for: Impact of panelists’ experience on script concordance test scores of medical students
Source: BMC Med Educ. 2020 Sep 17;20:313. doi: 10.1186/s12909-020-02243-w (PMC7499961; doi:10.1186/s12909-020-02243-w)
Supplement: Supplementary file 3 — Additional file 3. Cronbach’s alpha coefficients according to the experience, the specialty and the sample size of the panel of experts. [file 12909_2020_2243_MOESM3_ESM.pdf]

Additional file 3 Cronbach's alpha coefficients according to the experience, the specialty and the sample size of the panel of experts

	Residents			Non experienced physicians			Experienced physicians			Cardiologists			Emergency physicians		
	N=20	N=15	N=10	N=20	N=15	N=10	N=20	N=15	N=10	N=20	N=15	N=10	N=20	N=15	N=10
Cronbach's alpha	0.49	0.50	0.43	0.50	0.49	0.49	0.47	0.46	0.46	0.47	0.53	0.48	0.45	0.46	0.45
