# Supplementary material for: Impact of panelists’ experience on script concordance test scores of medical students
Source: BMC Med Educ. 2020 Sep 17;20:313. doi: 10.1186/s12909-020-02243-w (PMC7499961; doi:10.1186/s12909-020-02243-w)
Supplement: Supplementary file 4 — Additional file 4 SCT scores of the 935 medical students according to the level of experience (residents, non experienced and experienced physicians) and the size (N = 20, 15 and 10) of the panel of experts. [file 12909_2020_2243_MOESM4_ESM.pdf]

Additional file 4 SCT scores of the 935 medical students according to the level of experience (residents, non experienced and experienced physicians) and the size (N=20, 15 and 10) of the panel of experts

	Residents			Non experienced physicians			Experienced physicians		
	N=20	N=15	N=10	N=20	N=15	N=10	N=20	N=15	N=10
Median [IQR]	69.1 [63.6-74.7]	70.1 [64.8-75.8]	68.4 [62.7-73.7]	67.1 [60.6-73.3]	67.2 [60.6-73.3]	67.7 [61.3-73.8]	65.4 [59.6-70.8]	66.0 [60.2-71.3]	62.5 [57.0-68.3]
Mean (SD)	68.8 (8.7)	69.7 (8.7)	67.9 (8.6)	66.6 (9.4)	66.7 (9.3)	67.7 (9.6)	65.1 (8.6)	65.6 (8.6)	62.3 (8.6)
Min-max	26.8-89.4	27.6-90.6	27.4-90.6	24.4-90.5	23.4-89.3	26.1-91.7	27.7-85.5	26.0-87.2	26.7-85.1
