# Supplementary material for: Impact of panelists’ experience on script concordance test scores of medical students
Source: BMC Med Educ. 2020 Sep 17;20:313. doi: 10.1186/s12909-020-02243-w (PMC7499961; doi:10.1186/s12909-020-02243-w)
Supplement: Supplementary file 5 — Additional file 5. SCT scores of the 935 medical students depending on the specialties (cardiologists and emergency physicians) and the size (N = 20, 15 and 10) of the panel of experts. [file 12909_2020_2243_MOESM5_ESM.pdf]

Additional file 5 SCT scores of the 935 medical students depending on the specialties (cardiologists and emergency physicians) and the size (N=20, 15 and 10) of the panel of experts

	Cardiologists			Emergency physicians		
	N=20	N=15	N=15	N=20	N=15	N=10
Median [IQR]	66.4 [60.6-72.5]	68.3 [62.0-74.1]	68.9 [62.8-74.9]	67.3 [61.7-72.6]	70.2 [64.7-75.2]	68.9 [63.4-74.4]
Mean (SD)	66.1 (9.1)	67.9 (9.4)	68.5 (9.3)	66.9 (8.3)	69.6 (8.2)	68.5 (8.4)
Min-max	24.6-90.4	27.0-93.0	27.3-92.8	27.0-91.0	29.1-88.8	27.4-91.0
