# Supplementary material for: Impact of panelists’ experience on script concordance test scores of medical students
Source: BMC Med Educ. 2020 Sep 17;20:313. doi: 10.1186/s12909-020-02243-w (PMC7499961; doi:10.1186/s12909-020-02243-w)

Additional file 6 SCT scores depending on the number of traineeships performed by students in cardiology or emergency medicine

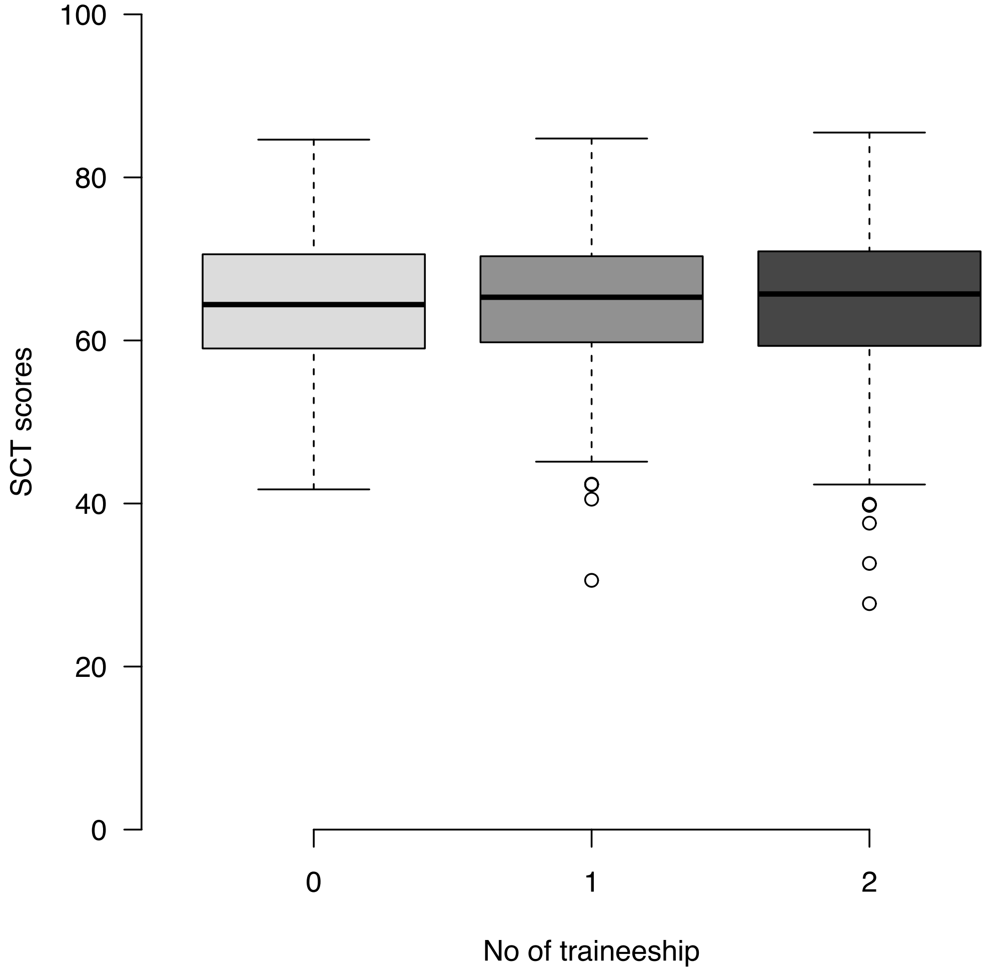

Supplement: Supplementary file 6 — Additional file 6. SCT scores depending on the number of traineeships performed by students in cardiology or emergency medicine. [file 12909_2020_2243_MOESM6_ESM.pdf]
